# Multiple burr hole surgery as a treatment modality for pediatric moyamoya disease

**DOI:** 10.4103/1817-1745.76102

**Published:** 2010

**Authors:** Ravindranath Kapu, Nigel Peter Symss, Goutham Cugati, Anil Pande, Chakravarthy M. Vasudevan, Ravi Ramamurthi

**Affiliations:** Dr. Achanta Lakshmipathi Neurosurgical Centre, Post Graduate Institute of Neurological Surgery, V.H.S Hospital, Chennai, India

**Keywords:** Children, indirect revascularization, moyamoya disease, multiple burr holes

## Abstract

**Objective:**

To re-emphasize that indirect revascularization surgery alone, where multiple burr holes and arachnoid openings are made over both cerebral hemispheres, is beneficial in the treatment of moyamoya disease (MMD) in children.

**Clinical Presentation:**

We report a 10-year-old boy who presented with complaints of episodic headache for the last 5 years. At the peak of his headache he had visual disturbances and acute onset weakness of left-sided limbs, recovering within a few minutes. He had no focal neurological deficits. Radiological investigations revealed abnormal findings, demonstrating the features of MMD.

**Surgical Management:**

He underwent bilateral multiple burr holes, dural and arachnoid opening over the frontal, parietal and temporal regions of each hemisphere. The elevated periosteal flap was placed in contact with the exposed brain through each burr hole.

**Results:**

On 6-months follow-up he had only one episode of transient ischemic attack. Postoperative four vessel angiogram demonstrated excellent cerebral revascularization around the burr hole sites, and single photon emission computerized tomography imaging showed hypoperfusion in the right temporo-occipital area suggestive of an old infarct with no other perfusion defect in the rest of the brain parenchyma.

**Conclusion:**

In children with MMD this relatively simple surgical technique is effective and safe, and can be used as the only treatment without supplementary revascularization procedures. This procedure can be done in a single stage on both sides and the number of burr holes made over each hemisphere depends on the extent of the disease.

## Introduction

Moyamoya, meaning a “hazy puff of smoke” in Japanese, was first described by Takeuchi and Shimizu in 1957.[1] It is a chronic, occlusive cerebrovascular disease involving bilateral stenosis or occlusion of the terminal portion of the internal carotid arteries (ICAs) and/or the proximal portions of the anterior cerebral arteries (ACAs), middle cerebral arteries (MCAs) and also the posterior circulation, with an abnormal vascular network at the base of the brain.[2] It is the most common pediatric cerebrovascular disease in Eastern Asia. The clinical presentation of moyamoya disease (MMD) in children usually includes episodes of transient ischemic attacks (TIAs), whereas in adults it is intracranial hemorrhage.[3,4] Very little is known about the pathogenesis of MMD, and thus curative treatment is still elusive. However, the benefits of revascularization surgery for the ischemic type of MMD are well-established.[5] Several surgical techniques have been described, in which revascularization of the ischemic regions of the brain is attempted. Direct revascularization by superficial temporal artery-middle cerebral artery (STA-MCA) anastomosis and indirect methods such as encephalomyosynangiosis (EMS), encephaloduroarteriosynangiosis (EDAS), encephaloduroarteriomyosynangiosis (EDAMS), omental graft have been performed with varying results[6–11] and a combination of direct and indirect revascularization techniques have also been attempted.[12–14] Most surgical procedures have aimed at increasing the blood supply primarily to the MCA territory and do not directly benefit the ACA territory.[4,12,15,16]

### Clinical presentation

*History and presentation:* This 10-year-old male child presented with complaints of chronic episodic headache of 5-years duration. Initially, at the peak of his headache he had only weakness of the left upper limb, but as his symptoms progressed he also developed weakness of the left lower limb, with speech and visual disturbances, lasting for a few minutes, followed by complete recovery. Six months prior to his admission he experienced four such episodes per day, with each attack of ischemia lasting for half an hour, followed by complete recovery. There was no history of loss of consciousness, or tonic clonic movements suggestive of a seizure. He was started on anticonvulsants elsewhere. There was no contributory family history.

#### Examination

On examination, the child was conscious, oriented with normal higher intellectual functions, with an intelligent quotient corresponding to his age. His fundi and cranial nerves functions were normal and he had no spinomotor or sensory deficits. His other systemic examination was normal.

#### Radiological investigations

**Figure 1:**
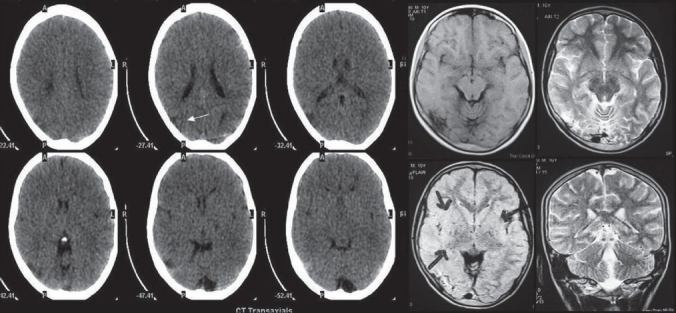
CT and MRI of the brain showing multiple lacunar infarcts bilaterally.

**Figure 2:**
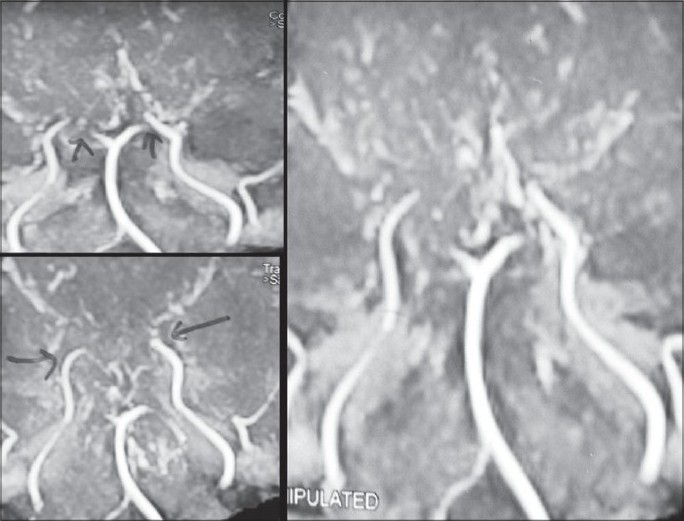
MR angiogram of the brain showing bilateral stenosis of the supraclinoid segment of the internal carotid artery and posterior circulation with multiple collaterals.

**Figure 3:**
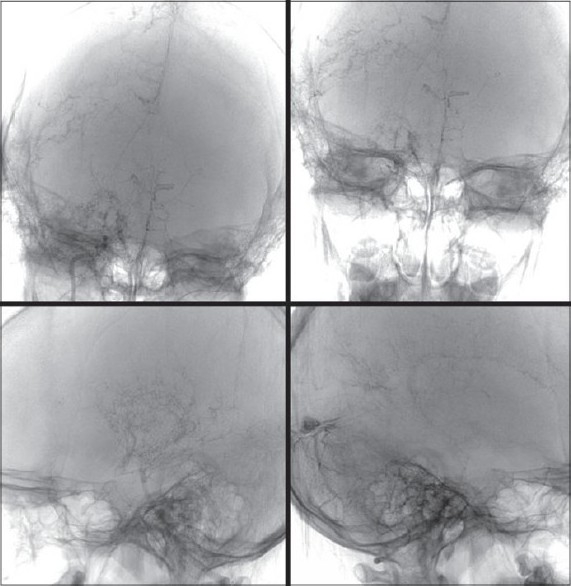
Digital subtraction four-vessel angiography showing bilateral multiple areas of stenosis of the supraclinoid portion of the internal carotid artery and the anterior cerebral, middle cerebral arteries and the posterior circulation with multiple collaterals arising proximal to the occluded vessels and from the external carotid artery

**Figure 4:**
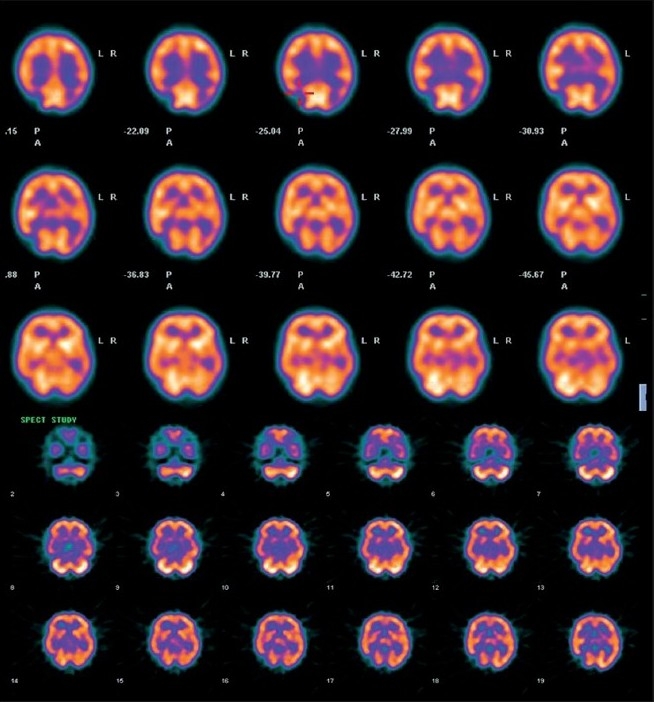
SPECT images of the brain showing hypoperfusion in the right temporo-occipital area suggestive of an old infarct, with other areas in brain parenchyma showing normal perfusion.

MRI and CT scan of the brain plain and contrast study showed multiple lacunar infarcts bilaterally [Figure 1]. MR angiogram of the brain showed bilateral stenosis of the ICA distal to the supraclinoid segment and in the posterior circulation [Figure 2]. Digital subtraction four-vessel angiography confirmed bilateral multiple stenosis of the supraclinoid portion of the ICA as well as stems of the ACA and MCAs and the posterior circulation with multiple collaterals arising proximal to the occluded vessels and from the external carotid artery [Figure 3]. Single photon emission computerized tomography (SPECT) images of the brain showed hypoperfusion in the right temporo-occipital area suggestive of an old infarct, with other areas in the brain parenchyma showing normal perfusion [Figure 4]. Adenosine brain SPECT study for evaluation of cerebrovascular reserve in stress and at rest showed a matched perfusion defect in the right temporo-occipital area, with no evidence of a new perfusion defect.

### Surgical technique

**Figure 5:**
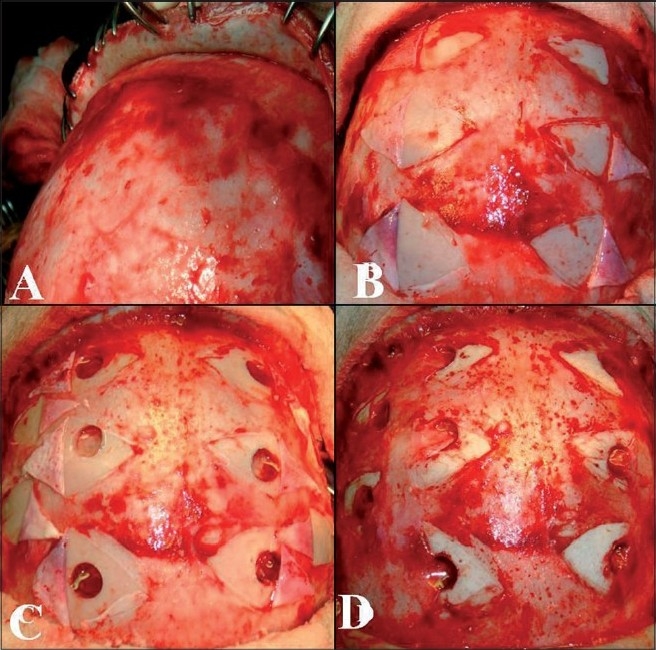
Intraop pictures showing multiple burr holes drilled over the exposed areas of bone, through small incisions in the perisoteum a) scalp elevation, b) periosteal elevation, c) burr holes and dural opening and d) periosteal flap placed in direct contact with the brain.

**Figure 6:**
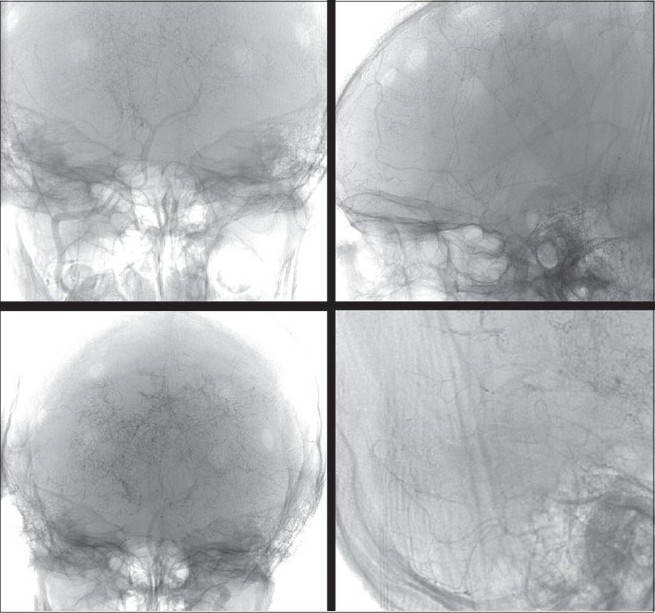
Postoperative four-vessel angiogram done after 6 months showing excellent cerebral revascularization around the burr hole sites.

**Figure 7:**
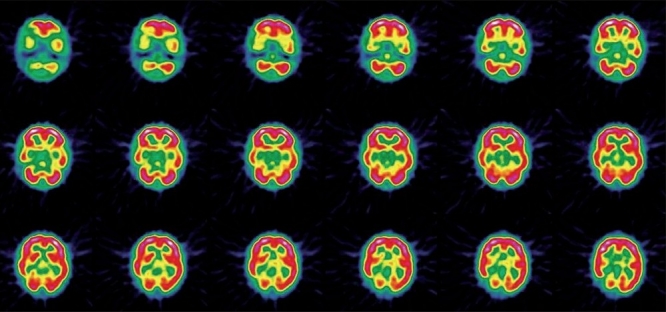
Postoperative SPECT imaging showing hypoperfusion in the right temporo-occipital area suggestive of an old infarct. There is evidence of no other perfusion defects seen in the rest of the brain parenchyma.

Under general anesthesia, with the patient in supine position, head kept neutral and flexed so that there is good exposure of the calvaria, a bicoronal skin flap was marked. The skin was infiltrated with saline solution in the subgaleal plane as this makes the dissection easier. The skip flap was turned taking care to preserve the galea aponeurotica and scalp blood supply. The periosteum was left attached to the bone, preserving the vessels as this will form the collateral network. Multiple triangular-shaped incisions were made in the periosteum and elevated as small flaps to expose the bone. Five burr holes were made on each side over the frontal, temporal and parietal region. The burr holes are made at each exposed area, using a high-speed drill, about 3 cm apart and 3 cm off the midline to prevent injury to the sagittal sinus. The surgical microscope is then used and the dura was opened through the burr holes, preserving the meningeal arteries. The arachnoid and the pia are then opened just enough to ensure a true opening without causing significant bleeding. Cautery was avoided to preserve the potential anastomotic vessels. The elevated periosteal flaps were laid over the exposed brain through the corresponding burr holes [Figure 5]. The galea was carefully replaced and the scalp was closed in two layers. A compressive head dressing was applied for 5 days. Postoperatively there was no cerebrospinal fluid (CSF) leak. The child is doing well and on 6-months follow-up had one episode of TIA. The postoperative effectiveness of the neovascularization was demonstrated 6 months later by four-vessel angiogram showing excellent cerebral revascularization around the burr hole sites [Figure 6] and SPECT imaging [Figure 7] showed hypoperfusion in the right temporo-occipital area suggestive of an old infarct. There were no other perfusion defects in the rest of the brain parenchyma.

## Discussion

Endo *et al*., in 1989 demonstrated marked neovascularization across a frontal burr hole, which was used for external drainage of an intraventricular hemorrhage in a child with MMD.[12] On the basis of this, they used additional frontal burr holes with EMS for treating children with MMD, and found better revascularization of the frontal area when compared to EMS alone. Kawaguchi *et al*,[17] performed the burr hole procedure alone in 1 adult and Sainte-Rose *et al*,[18] used the similar procedure of indirect revascularization in 14 children. They both demonstrated excellent revascularization without the necessity of a supplementary procedure.

Even though the pathogenesis of MMD remains unclear, there are prominent pathological features noted; proliferation and migration of smooth muscle cells in the intima, leading to intimal thickening with morphological and biochemical alteration of extracellular matrix components such as elastin, collagen and proteoglycans[2,19] without signs of any inflammation or atheromatous plaque.[1,20] Junichi *et al*,[21] found MMD to have a bimodal peak of distribution with the first peak at 10 years and the second in the fourth decade. Clinically our patient had a history of multiple episodes of TIA, with radiological evidence of an area of chronic infarction which categorized him to be in stage IV Matsushima clinical grading of MMD.[9] Based on the angiographical findings, the patient was in Suzuki and Takaku stage IV; he had progressive narrowing of all the main cerebral arteries with almost complete disappearance of the posterior cerebral artery with minimal residual patency of basal vessels and the development of extracranial collateral vessels.[2,22] SPECT scan is the most commonly used test for evaluation of the hemodynamic changes in MMD and the finding of hypoperfusion generally correlates well with the clinical symptoms.[23] Preoperatively we did an adenosine brain SPECT study for evaluation of cerebrovascular reserve in stress and at rest. Adenosine infusion causes vasodilatation of cerebralarteries and can be used for the investigation of cerebrovascularperfusion capacity in patients with carotid occlusive disease. One advantage in the use of adenosine over acetazolamide isthe possibility of interrupting the test with reversal of clinicalsymptoms or patient discomfort within a few minutes.[24]

The surgical procedures performed for ischaemic type of MMD can be classified into three categories: *direct bypass, indirect bypass and combined revascularization techniques*. The results obtained have been reported to be excellent, but it is still not clear which technique is the safest and most effective. Direct procedure includes STA-MCA anastomosis. This procedure was first adopted by Yasargil and repeated by Karyenbuhl.[25] The early improvement of cerebral hemodynamics is considered to be one of the major advantages of this procedure.[26,27] However, the clinical outcome is not different from that achieved by an indirect procedure, thus making its benefit questionable. Also this procedure has limitations in the treatment of pediatric MMD, due to the small size of the donor and recipient vessels and the need for temporary occlusion of blood flow in the cortical artery during anastomosis.[15,16] The indirect methods described are EMS, EDAS, EDAMS, omental flaps and pial synangiosis[6–9] and combinations of direct and indirect revascularization surgeries have also been done.[12–14] The principle of indirect revascularization in patients with MMD is based on the natural ability of collateral vessels to develop in these patients and, that revascularization of the ischemic brain areas can be achieved from an extracranial blood supply. Indirect procedures bring in circulation to the intracranial regions by introducing newly developed vasculature from newly approximated tissues.[28] Although not a specific marker for moyamoya, elevated basic fibroblast growth factor (bFGF) in CSF may serve as a weak predictor of the extent of angiogenesis to be expected in indirect revascularization procedures.[29] Yoshimoto *et al*, analyzed CSF samples obtained in patients with MMD and found changes in the CSF cytokines that act in an angiogenic manner.[30]

Based on the child’s clinical and radiological findings we decided to do an indirect revascularization surgery, by making multiple burr holes and arachnoid openings over both cerebral hemispheres. Five burr holes were made in the frontotemporoparietal area of each hemisphere, as the child had symptoms of TIA, causing leg and hand weakness, with visual disturbances. The number of burr holes made depends on the site and extent of the disease. Sainte-Rose *et al*,[18] from their study have demonstrated an early improvement in cerebral perfusion following placement of multiple burr holes and use this technique in all patients with MMD.

In children with MMD undergoing indirect revascularization techniques, or combined procedures, the extent of revascularization is dependent on the area and size of contact between the extracranial tissue and the brain, and Houkin *et al*, conclude that as much brain as possible be exposed and the external tissues be applied as widely as possible.[13] STA-MCA and EMS procedures improve symptoms mostly related to the MCA territory. They did not improve symptoms of TIA with leg weakness, abnormal cognition, and visual field defects due to ischemia in the areas supplied mainly by the ACA and posterior cerebral artery.[6,31,32] Also, it is well known that MMD in children is dynamic and progressive,[3] thus deterioration of blood flow in the ACA territory can progress despite good collateral formation in the MCA territory.

## Conclusion

This technique described can be performed in a single stage, on both sides by using a bicoronal flap, where multiple burr holes can be placed over the whole hemisphere where necessary. The procedure is short and safe, with excellent revascularization of most of the cortex underlying the burr holes, and additional revascularization surgeries are not required. The duration is short-making it an option in high-risk cases.
